# Supplementary material for: Mechanism of Cadmium Exposure Induced Hepatotoxicity in the Mud Crab (Scylla paramamosain): Activation of Oxidative Stress and Nrf2 Signaling Pathway
Source: Antioxidants (Basel). 2022 May 17;11(5):978. doi: 10.3390/antiox11050978 (PMC9137997; doi:10.3390/antiox11050978)
Supplement: Supplementary file 1 [file antioxidants-11-00978-s001.zip › antioxidants-1696894-supplementary.pdf]

Table S1 Primer sequences of all genes used in this study.

Primers name	Nucleotide sequence (5´-3´)
RT-18S-F	TTTTCTGAACCCGAGGTAATGAC
RT-18S-R	ATGCTTTCGCAGTAGTTCGTCTT
RT-Nrf2-F	AGTGTCAGGGTCTTCTGTGGAGC
RT-Nrf2-R	CATAGGCAGGTTGATGATGTCGT
RT-CYP2-F	TGGTGGCAAGCAGACAGTTCG
RT-CYP2-R	GCCTTGTGCTTCTCGGTCTCAT
RT-MT-F	CAAATGTGCCAACAAGGAGGA
RT-MT-R	TGTGACGGTGGGGAATGATG
dsNrf2-F(with T7)	TAATACGACTCACTATAGGGCCGTTGGA CAGATACAGGT
Nrf2-R	CCTCATCAGCCAGGTGTGTA
dsNrf2-R(with T7)	TAATACGACTCACTATAGGCCTCATCAG CCAGGTGTGTA
Nrf2-F	GCCGTTGGACAGATACAGGT
dsGFP-F(with T7)	TAATACGACTCACTATAGGATGGTGAGC AAGGGCGAGGAG
dsGFP-R	ATGGTGAGCAAGGGCGAGGAG
dsGFP-R(with T7)	TAATACGACTCACTATAGGTCAAAGATC TACCATGTACAGCTCGT
dsGFP-R	TCAAAGATCTACCATGTACAGCTCGT

Figure S1. IBRv2 values following biomarkers (SOD, CAT, MDA, T-AOC and H_2O_2) in a gradient of cadmium exposure affection. (A) 0.01 mg/L cadmium treatment group; (B) 0.05 mg/L cadmium treatment group; (C) 0.125 mg/L cadmium treatment group. IBRv2 values were compared to the control group.

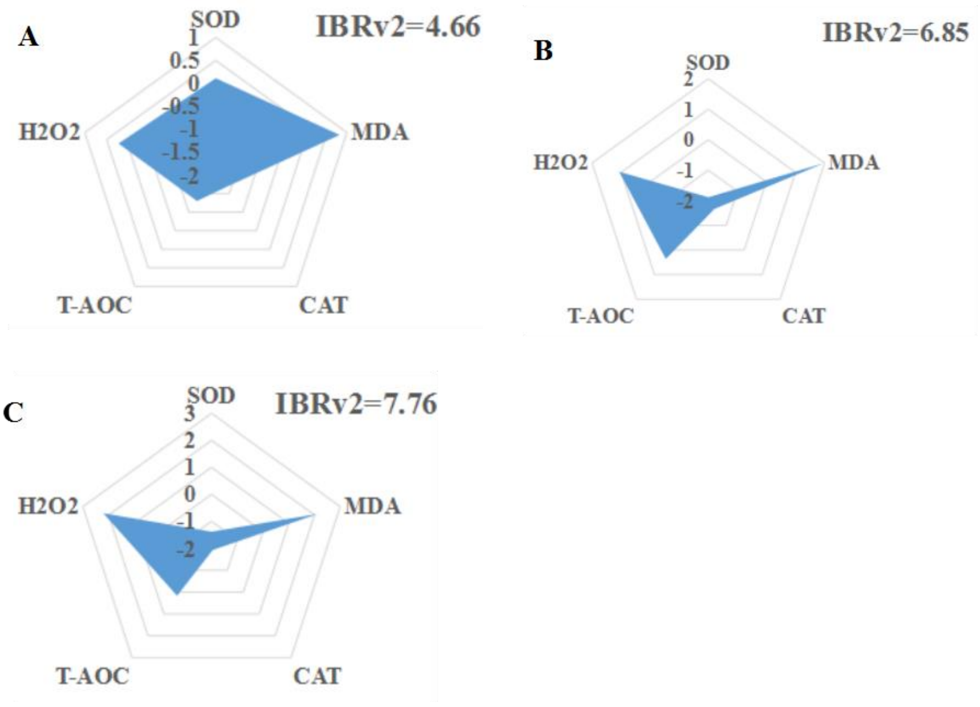

Abbreviations

SOD: Superoxide Dismutase; ***H_2O_2:***Hydrogen Peroxide; ***CAT:*** Catalase; ***MDA:***Malondialdehyde; ***GST:***Glutathione S-transferase; ***ROS:***Reactive Oxygen Species; ***CYPs:***Cytochrome P450 enzymes; ***MT:***Metallothionein; ***T-AOC:***Total Antioxidant Capacity; ***qRT-PCR:***Quantitative real time PCR; ***GFP:***Green Fluorescent Protein; ***IBRv2:*** Integrated biological responses version 2.
